# Supplementary material for: Causal Relationship between Aging and Anorexia Nervosa: A White-Matter-Microstructure-Mediated Mendelian Randomization Analysis
Source: Biomedicines. 2024 Aug 16;12(8):1874. doi: 10.3390/biomedicines12081874 (PMC11351342; doi:10.3390/biomedicines12081874)
Supplement: Supplementary file 1 [file biomedicines-12-01874-s001.zip › Supplementary Figure/Figure S8/Leaveoneout plot of MR analysis for TL to ubm-b-2028.pdf]

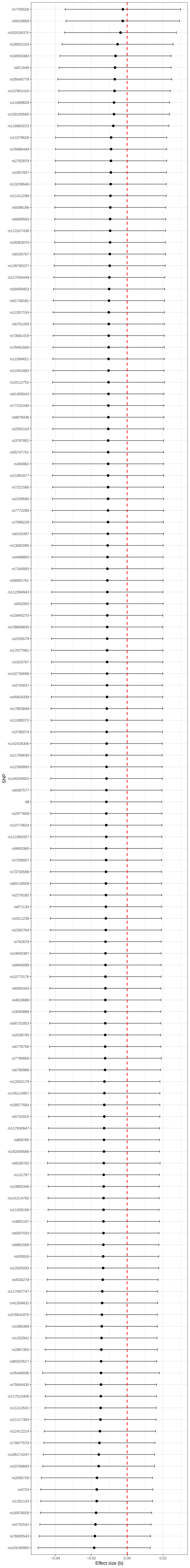

rs7705526
rs9419958
rs202034370
rs28502153
rs34550383
rs611646
rs35640778
rs137901416
rs11699829
rs150150565
rs116863223
rs11579626
rs76666449
rs2763979
rs1957937
rs13230646
rs11412296
rs9398196
rs6669563
rs111527438
rs28363070
rs8105767
rs139795227
rs117034449
rs59409453
rs61748181
rs11557154
rs6751209
rs73581419
rs78491606
rs11584821
rs12451892
rs10112752
rs61405042
rs77231040
rs9878436
rs2555104
rs3767952
rs55747751
rs450962
rs11991877
rs7221585
rs2230590
rs7772289
rs7099229
rs8102497
rs13062095
rs4498805
rs7164950
rs56061761
rs112394943
rs932002
rs10840270
rs139669835
rs2293579
rs17677991
rs1023767
rs142730696
rs4743037
rs45604339
rs17803849
rs11085072
rs3785074
rs142426306
rs11769630
rs12369950
rs144204502
rs6587577
All
rs2977608
rs10774624
rs111950327
rs9955360
rs7209057
rs73730598
rs80116508
rs2276182
rs871134
rs1611236
rs2282764
rs762679
rs10845387
rs9940099
rs10773176
rs6590343
rs4616688
rs3093888
rs66731853
rs2538745
rs6776756
rs7790856
rs6790988
rs12932179
rs145114957
rs28577594
rs5742915
rs117630647
rs869785
rs182059586
rs6536702
rs131797
rs10805346
rs141214782
rs11426156
rs3891167
rs6007020
rs6881568
rs939916
rs12925933
rs4530278
rs117407747
rs41304832
rs376641875
rs1985369
rs1332941
rs2967355
rs80324517
rs35446936
rs75664430
rs117512405
rs11212631
rs11117354
rs12412214
rs79977579
rs185174247
rs10768683
rs2056726
rs4724
rs1291143
rs16978028
rs4731541
rs76065543
rs143190905
SNP
−0.04
−0.02
0.00
0.02
Effect size (b)
